# Hypercalcemia Secondary to Calcitriol Production From Dual Combination Immunotherapy in Pulmonary Metastatic Melanoma

**DOI:** 10.7759/cureus.62379

**Published:** 2024-06-14

**Authors:** Andrew Strike, Steven B Barker, Nicole McGuire, Gurleen Kaur

**Affiliations:** 1 Graduate Medical Education, Northeast Georgia Medical Center Gainesville, Gainesville, USA; 2 Internal Medicine, Northeast Georgia Medical Center Gainesville, Gainesville, USA

**Keywords:** malignancy, sarcoidosis, macrophage activation, immune-related adverse events, ipilimumab, nivolumab, metastatic melanoma, dual combination immunotherapy, calcitriol, hypercalcemia

## Abstract

Nivolumab and ipilimumab are immunotherapy agents recommended for the treatment of metastatic melanoma. A rare adverse effect of these agents is hypercalcemia. The mechanism of immunotherapy-mediated hypercalcemia is thought to be due to ectopic calcitriol production from activated macrophages, similar to sarcoidosis. We present a case of a 76-year-old female with metastatic melanoma who developed severe hypercalcemia after completing a cycle of combined nivolumab and ipilimumab therapy. After other common causes of hypercalcemia in malignancy were ruled out, the decision was made to aggressively treat her hypercalcemia while inpatient and hold immunotherapy at discharge. Since holding immunotherapy, she has not had a repeat occurrence of hypercalcemia. This case stresses the importance of including immunotherapy adverse effects in the differential diagnosis for hypercalcemia in malignancy.

## Introduction

Hypercalcemia is a common metabolic complication of malignancy, affecting up to 30% of cancer patients [1]. It can result from numerous pathologies, including ectopic production of calcitriol. Dual immunotherapy with nivolumab and ipilimumab has been shown to improve survival in metastatic melanoma patients but has also been associated with immune-related adverse events, including hypercalcemia [2]. The National Comprehensive Cancer Network endorses the use of dual immunotherapy, specifically with nivolumab and ipilimumab, for treating metastatic melanoma [3]. Nivolumab functions as an anti-programmed death 1 (PD1) monoclonal antibody that selectively blocks immune checkpoint receptors on activated T cells, amplifying the anti-tumor immune response. Ipilimumab specifically binds to cytotoxic T-lymphocyte-associated protein 4 (CTLA-4) receptors located on T-lymphocytes, obstructing interaction with CD86 and CD80 ligands, which increases T-cell activation [2,3].

## Case presentation

**Figure 1:**
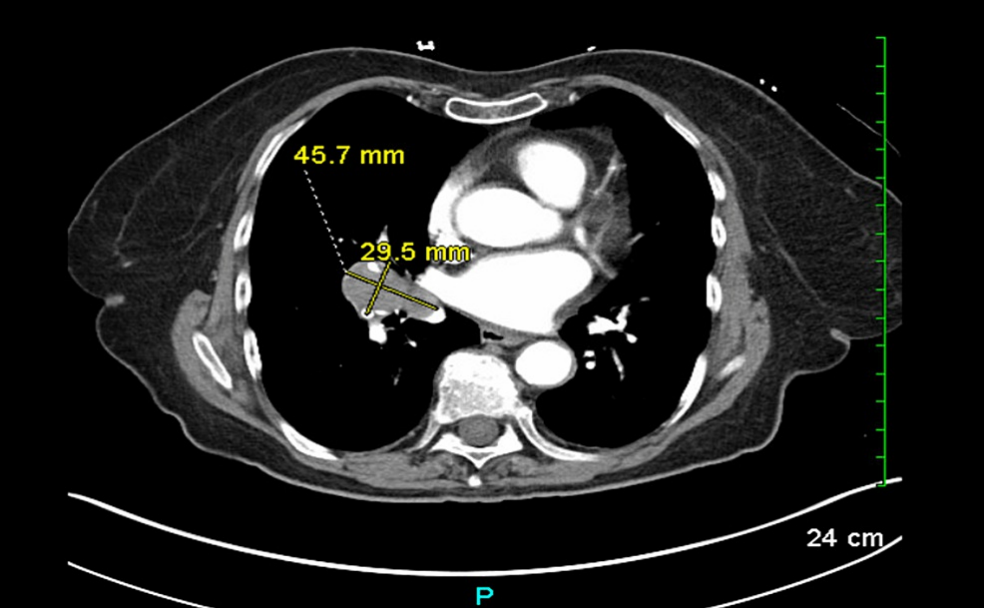
Computed tomography scan of the patient's chest, showing the newly identified lung mass

A 76-year-old female with a history of stage II melanoma, status post-Mohs procedure nearly 10 years ago, and tobacco use disorder with a 20-pack-year smoking history presented to the emergency department (ED) with an intractable headache. Incidental lung nodules were identified on the chest X-ray. Subsequent computed tomography imaging confirmed the presence of a 4.6 x 3.0 cm lung mass (Figure 1).

The patient was referred to pulmonology after discharge from the ED. Outpatient bronchoscopy with biopsy revealed metastatic melanoma with positive SOX10, Melan-A, S100, and HMB45 tumor markers. These findings were most consistent with metastatic melanoma. The patient was started on dual immunotherapy with intravenous (IV) nivolumab 1 mg/kg and ipilimumab 3 mg/kg once every three weeks; she was scheduled for four total cycles. After completing her second treatment cycle, she returned to the ED with symptoms of weakness, body aches, shortness of breath, nausea, vomiting, and decreased appetite. On presentation, her vitals were as follows: blood pressure 160/90 mmHg, heart rate 110 bpm, respiratory rate 22, and temperature 98.9°F. She was alert and oriented to self and location only while also experiencing hypersomnolence with intermittently confused responses to questioning. Laboratory findings revealed a corrected calcium level of 15.1 mg/dL. Due to symptomatic hypercalcemia, the patient was admitted to the hospitalist service, and further work-up was completed. Laboratory testing was significant for suppressed parathyroid hormone (PTH) level at 9 pg/mL, undetectable parathyroid hormone-related peptide (PTHrP) at <0.4 mmol/L, normal 25-hydroxyvitamin D level at 53 ng/mg, and markedly elevated calcitriol level at 106 pg/mL. Serum and urine protein electrophoresis did not show any abnormal findings. The serum-free light chain assay did not show an elevated kappa/lambda ratio, and immunofixation electrophoresis showed no monoclonal immunoglobulins. Further imaging did not reveal signs of sarcoid-like granulomatous disease. A careful review of recent imaging also did not include any lytic lesions or bony abnormalities. After a review of cases with similar presentations, the suspected etiology was calcitriol-mediated hypercalcemia due to immunotherapy. The immunotherapy regimen was temporarily withheld, and the patient was treated with calcitonin, IV dexamethasone, and IV fluids, leading to the normalization of calcium levels. Once the patient’s symptoms resolved, she was discharged with close follow-up by oncology. Of note, since stopping immunotherapy, she has not had a repeat episode of hypercalcemia.

## Discussion

This case illustrates a rare occurrence of calcitriol-mediated hypercalcemia secondary to dual immunotherapy. While multiple etiologies for hypercalcemia exist in malignancy, calcitriol-mediated hypercalcemia was the most plausible explanation, given the elevated calcitriol levels and the absence of other common causes. The current hypothesis is that increased macrophage activation caused by immunotherapy may lead to increased production of a specific 25(OH)D-hydroxylase enzyme, which is nonresponsive to PTH [4]. Previous case reports have documented similar events with nivolumab and ipilimumab, with some cases requiring corticosteroid use for symptom resolution [5-7]. To our knowledge, this is the first documented case of immunotherapy-mediated hypercalcemia in the setting of metastatic melanoma. Corticosteroids inhibit the conversion of vitamin D and increase urinary calcium loss, contributing to the resolution of hypercalcemia [8].

Nivolumab, an anti-PD1 monoclonal antibody, works by selectively blocking immune checkpoint receptors on activated T cells, amplifying the antitumor immune response. Conversely, ipilimumab binds to CTLA-4 receptors on T-lymphocytes, blocking interaction with CD86 and CD80 ligands and increasing T-cell activation. These mechanisms enhance the body's immune response against cancer cells but can also lead to immune-related adverse events, such as hypercalcemia. The pathophysiology of hypercalcemia in cancer patients typically involves increased bone resorption, increased renal reabsorption of calcium, or increased intestinal absorption of calcium [8]. Calcitriol, the active form of vitamin D, promotes calcium absorption in the intestines and increases renal calcium reabsorption [8]. The markedly elevated calcitriol levels in this patient, in the absence of other causes, point to ectopic production of calcitriol as the underlying cause of hypercalcemia. Further investigation into the patient's medical history revealed no prior episodes of hypercalcemia or granulomatous diseases such as sarcoidosis, which are known to cause increased calcitriol production [9]. The absence of bone metastases on imaging ruled out local osteolytic hypercalcemia. The patient's suppressed PTH and undetectable PTHrP levels effectively ruled out primary hyperparathyroidism and humoral hypercalcemia of malignancy [10]. Table 1 gives an overview of common causes of hypercalcemia in malignancy, along with evidence from the clinical case that supported our diagnosis [9-11]. Given the timeline of symptom onset following the administration of dual immunotherapy, it was concluded that the hypercalcemia was immune-related.

**Table 1:** Overview of common causes of hypercalcemia in malignancy, with the patient's clinical history and conclusions regarding diagnosis PTH: parathyroid hormone; PTHrP: parathyroid hormone-related peptide

Cause of hypercalcemia	Evidence from history	Conclusion
Humoral hypercalcemia of malignancy	PTHrP: <0.4 mmol/L (undetectable)	Ruled out due to undetectable PTHrP level
Local osteolytic hypercalcemia	No mention of bone metastases or skeletal involvement	Ruled out due to absence of bone metastases
Ectopic production of PTH	PTH: suppressed at 9 pg/mL	Ruled out due to suppressed PTH level
Ectopic production of calcitriol (typically due to sarcoidosis, malignancy)	Calcitriol: elevated at 106 pg/mL. No signs of granulomatous disease on imaging or history. No signs of other malignancy aside from metastatic melanoma	Likely caused due to elevated calcitriol. However, melanoma-producing ectopic calcitriol is exceedingly rare

## Conclusions

This case stresses the importance of recognizing the adverse effects of immunotherapy, like hypercalcemia. As this medication regimen is common in patients with metastatic melanoma, rare side effects need to be considered by the physician team managing this complicated patient population. In our case, a comprehensive diagnostic evaluation ruled out other common causes of hypercalcemia, such as primary hyperparathyroidism, local osteolytic lesions, and humoral/PTHrP-mediated hypercalcemia of malignancy. The lack of radiographic findings or noncaseating granulomas also made sarcoidosis unlikely. Markedly elevated calcitriol levels without other typical causes support an immunotherapy-related mechanism. After aggressive treatment for hypercalcemia and cessation of immunotherapy, calcium levels normalized, and there were no further episodes of hypercalcemia during subsequent outpatient visits.
